# Sleep Duration and Diabetic Kidney Disease

**DOI:** 10.3389/fendo.2018.00808

**Published:** 2019-01-14

**Authors:** Nicholas Y. Q. Tan, Joel Chan, Ching-Yu Cheng, Tien Yin Wong, Charumathi Sabanayagam

**Affiliations:** ^1^Singapore National Eye Centre, Singapore Eye Research Institute, Singapore, Singapore; ^2^Department of Ophthalmology, Yong Loo Lin School of Medicine, National University of Singapore, Singapore, Singapore; ^3^Ophthalmology and Visual Sciences Academic Clinical Program, Duke-NUS Medical School, Singapore, Singapore

**Keywords:** diabetes, diabetic kidney disease, diabetic nephropathy, sleep, sleep duration

## Abstract

**Aims:** Abnormally short or long durations of sleep have been proposed as a risk factors for diabetes and its micro- and macro-vascular complications. However, the relationship between sleep duration and diabetic kidney disease (DKD) has not been well-characterized. Thus, we aimed to examine the association of sleep duration with DKD in two Asian populations.

**Methods:** We included 1,258 persons (Malay, *n* = 403; Indian, *n* = 855) aged 40–80 years with diabetes from a population-based cross-sectional sample from Singapore. DKD was defined by low estimated glomerular filtration rate (eGFR <60 mL/min/1.73 m^2^) and albuminuria (urinary albumin-to-creatinine ratio ≥30 mg/g, only measured in Indian participants). Self-reported habitual sleep duration was categorized into 4 categories: very short (<5 h), short (5–6.9 h), normal (7–8 h) and long (>8 h). The associations of sleep duration with low eGFR and albuminuria were analyzed using multivariable logistic regression models adjusted for multiple potential confounders (including classic risk factors such as HbA1c and hypertension).

**Results:** In total, 268 (21.3%) participants had low eGFR, and 271 (34.7% in Indians) had albuminuria. The number (%) of individuals with very short, short, normal, and long durations of sleep were 117 (9.3%), 629 (50.0%), 429 (34.1%), and 83 (6.6%), respectively. Long sleep duration was associated with a higher odds of renal insufficiency compared to normal sleep duration (OR [95% CI]: 2.31 [1.27–4.19]) on multivariable analysis. Similarly, both long and very short durations of sleep were associated with higher odds of albuminuria (OR [95%]: 2.44 [1.36, 4.38] and 2.37 [1.25, 4.50], respectively) in Indian participants (where data on albuminuria were available).

**Conclusions:** Our study suggests that abnormally short or long durations of sleep were associated with DKD, manifesting as either a reduced eGFR or increased albuminuria. However, further longitudinal data would be required for confirmation.

## Introduction

Diabetes is a growing public health challenge, with the projected number of individuals with diabetes expected to increase to 592 million in 2035, and the prevalence in Asia increasing at a much faster rate than in Western countries (1, 2). Approximately half of all patients with diabetes will develop diabetic kidney disease (DKD), which is clinically defined by the presence of impaired renal function or elevated urinary albumin excretion, or both (3–6). DKD is associated with considerable morbidity and mortality (7, 8). Furthermore, the progression of DKD to end-stage renal failure frequently requires renal replacement therapy, which carries large economic costs (9). Thus, it is imperative from both clinical and public health perspectives, to uncover additional modifiable risk factors for DKD in addition to the classic risk factors such as poor glycemic control and hypertension (10).

Sleep is a primordial behavior shared by all humans and serves a host of functions from the cellular to organismal level. In recent years, it is becoming clear that poor sleeping patterns place individuals at risk for a broad range of diseases including diabetes (11, 12). In a meta-analysis of prospective studies, both long and short durations of sleep were shown to be associated with higher risk of diabetes (13). Microvascular and macrovascular complications of diabetes, such as diabetic retinopathy and cardiovascular disease, respectively, have also been linked to abnormal durations of sleep (14, 15). However, literature on the relationship between sleep duration and DKD, an important microvascular complication of diabetes is scarce (15, 16). Two cross-sectional studies, one from Japan and the other from China have shown inconsistent findings. In the Japanese study, while long sleep duration (≥8.5 h) was associated with DKD in the China study, only short sleep duration (<6 h) was associated with DKD. Further studies are therefore required to validate the consistency of these reported associations in other populations. In Asia, Malays and Indians are the two ethnic groups that are at high risk for diabetes, in addition, Malays are also at higher risk for developing CKD (17). In this context, we examined the association of sleep duration with DKD in a population-based sample of Malay and Indian adults with diabetes in Singapore.

## Methodology

### Study Population

The Singapore Malay Eye Study (SiMES, 2004–07, *n* = 3280) and the Singapore Indian Eye Study (SINDI, 2007–09, *n* = 3,400) are two independent population-based cohort studies of Malay and Indian adults in Singapore. Follow-up visits were conducted from 2011 to 2015. The detailed methodology of SiMES-2 (18) and SINDI-2 (19) have been reported elsewhere. In brief, the sampling frame composed of all Indians and Malays aged 40–80 living in designated study areas in the south-western part of Singapore. From a list of names provided by the Ministry of Home Affairs, an age-stratified random sampling was used, and 4,168 Malay and 4,497 Indian subjects were determined to be eligible. Of these, 1,901 Malay participants (72.1% of eligible participants) and 2,200 Indian participants (75.5% of eligible participants) attended the SiMES-2 and SINDI-2 studies, respectively. As part of the objective of SiMES and SINDI to investigate the risk factors for various eye diseases, sleep-related data and measures of renal dysfunction were also collected as part of the study protocol. However, sleep-related data were collected only in the follow-up studies and not at baseline. Hence, for the current study, we used the cross-sectional data from the follow-up visit only. Both studies followed similar methodology and were conducted in the same study clinic. Written informed consent was obtained from all participants. The study adhered to the tenets of the declaration of Helsinki, and ethics committee approval was obtained from the SingHealth Centralized Institutional Review Board. In total, 1,901 Malay participants (72.1% of eligible participants) and 2,200 Indian participants (75.5% of eligible participants) attended the SiMES-2 and SINDI-2 studies, respectively.

**Figure 1:**
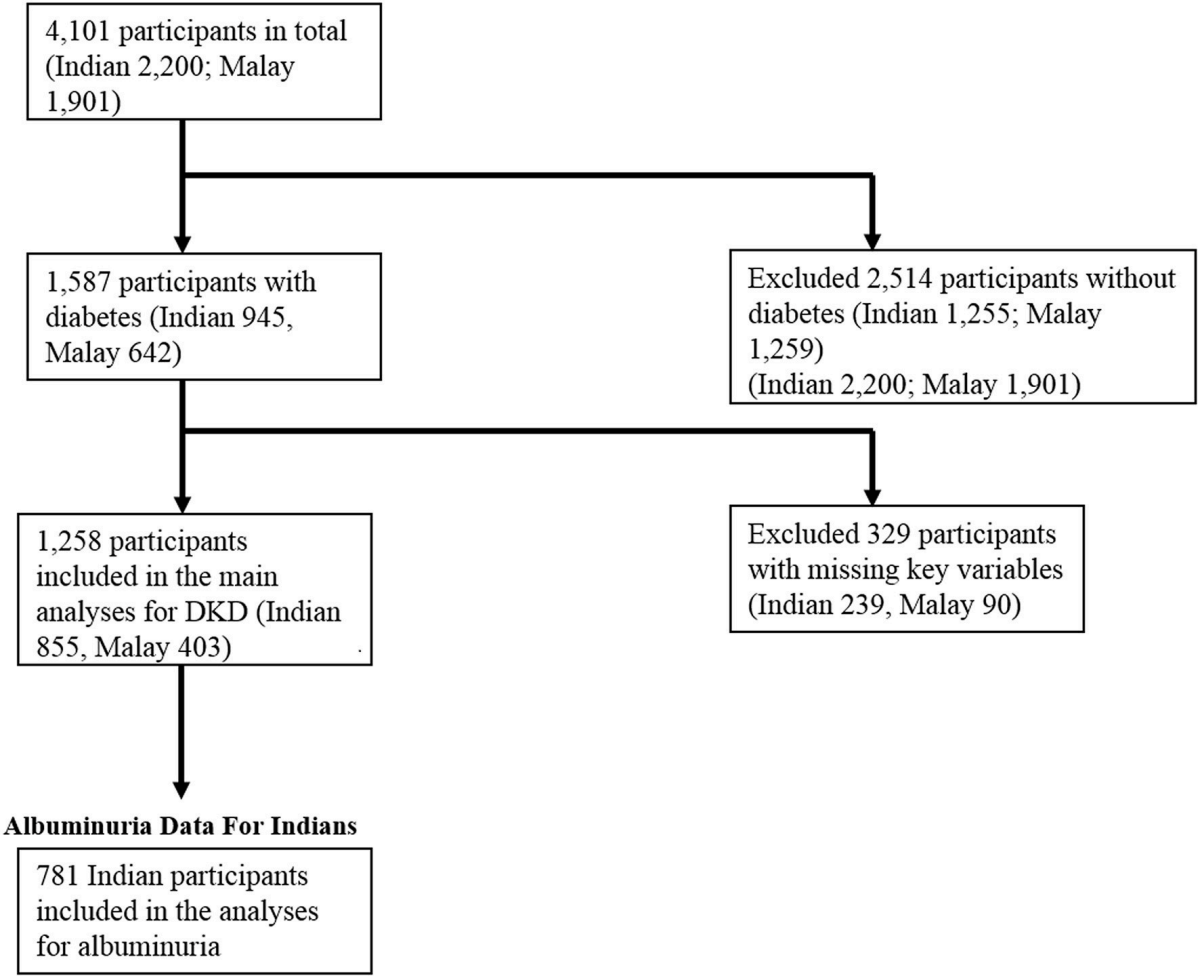
Flowchart of the inclusion and exclusion of participants for this study.

Of the total 4,101 participants in SIMES-2 and SINDI-2, 1,587 (38.7%) had diabetes. Of these, 274 participants had missing data on key variables (sleep duration or eGFR), and a further 50 participants had missing data on variables included in our multivariable models. Therefore, a total of 1,263 participants were included in our main analysis. Data for albuminuria were only available in the Indian participants, of which *n* = 74 had missing data. Therefore, only 781 Indian participants were included in a secondary analysis for albuminuria. A flowchart summarizing these inclusion and exclusion criteria is included in Figure 1.

### Assessments of Diabetic Kidney Disease

Venous blood samples were collected for measurement of serum glucose, HbA1c, and creatinine levels, and spot untimed urine samples were collected for measurement of albumin and creatinine. Diabetes was defined as random plasma glucose ≥200 mg/dl (11.1 mmol/l), HbA1c ≥6.5%, self-reported use of diabetic medication, or physician-diagnosed diabetes (20). Lower estimated glomerular filtration rate (eGFR) and albuminuria were utilized as measures of DKD in our study. Venous blood samples were collected for serum creatinine levels, and spot untimed urine samples were collected for measurement of albumin and creatinine. The eGFR was calculated from serum creatinine using the CKD-EPI equation (21). For our primary outcome, low eGFR was defined as eGFR <60 mL/min/1.73 m^2^. For our secondary outcome, albuminuria (only available in Indian participants) was defined as urinary albumin-to-creatinine ratios (UACR) of ≥30 mg/g (22).

### Assessment of Sleep Duration

Data on self-reported habitual sleep duration was collected using a standardized interviewer-administered questionnaire, conducted in English, Malay or Tamil according to the participant's preference. Participants were asked: “on average, how much sleep do you get in a 24-h day?” Self-reported habitual sleep duration reported to the nearest half-hour, and the duration of sleep was organized into 4 categories: very short (<5 h), short (5–6.9 h), normal (7–8 h), and long (>8 h). The normal duration of sleep was defined as 7–8 h according to the National Sleep Foundation recommended sleep time duration for older adults (23). Moreover, the reference range of 7–8 h has also been widely used in other epidemiological studies such as the Rotterdam Study and the Quebec Family Study (24, 25).

### Other Assessments

Information on participants' demographic characteristics and medical history was obtained by using a standardized questionnaire administered by trained personnel. This included educational level, respiratory disorders such as asthma and chronic obstructive pulmonary disease, use of medication for diabetes or hypertension, lifestyle factors such as current cigarette smoking, and mood related complaints. Educational level was recorded as the highest number of years of schooling completed and was categorized into 2 groups: (1) primary school level or lower (≤6 years) and (2) secondary school level or higher (≥7 years). Mood-related complaints were assessed using the question: “are you anxious or depressed?” Participants who reported being at least moderately anxious or depressed were considered to have mood-related complaints.

Blood pressure was measured using a digital automatic blood pressure monitor (Dinamap model Pro100V2; Criticon GmbH, Norderstedt, Germany) following the protocol used in the Multi-Ethnic Study of Atherosclerosis (26). Hypertension was defined as a systolic BP ≥140 mmHg, diastolic BP ≥90 mmHg, or use of antihypertensive medication (26). Height was measured using a wall-mounted tape, and weight with a digital scale (SECA, model 782 2321009; Vogel & Halke, Hamburg, Germany). Body mass index was defined as weight divided by the height in meters squared (kg/m^2^). Obesity was defined as BMI of ≥27.5 kg/m^2^ according to the recommended WHO Asian BMI cut-off points (27). Venous blood samples were also measured for random plasma glucose, hemoglobin A1c (HbA1c) and serum cholesterol levels.

### Statistical Analysis

Statistical analysis was performed using R 3.3.1 statistical computing language (R Core Team, 2016). The Chi-Square Test (for categorical variables) and ANOVA (for continuous variables) were then used to compare the demographic and clinical characteristics of participants between the 5 categories of sleep duration. Logistic regression models were used to assess the associations of very short, short, and long sleep durations (referenced against normal sleep duration with low eGFR. In a first model, odds ratios (ORs) and their 95% confidence intervals [95% CIs] were adjusted for age, gender and ethnicity. In a second multivariable model, additional adjustments were made for HbA1c, duration of diabetes, systolic blood pressure, and other potential confounders. We stratified results according to ethnicity in further sensitivity analyses. In a separate analysis including Indian participants only, the association of sleep duration with albuminuria was determined using similar logistic regression models. *P*-value for significance was set at <0.05.

## Results

A total of 1,258 participants with diabetes were included in the analyses; 403 (32.0%) were Malay and 855 (68.0%) were Indian; the mean age was 64.6 ± 9.1 years, and 640 (50.9%) were female. Of these, 133 (33.0%) Malay and 135 (15.8%) Indian participants hadlow eGFR. Of the Indian participants (with data on UACR), 271 (34.7%) had albuminuria. The mean duration of sleep was 6.4 ± 1.5 h. The demographic and clinical characteristics of the study participants stratified by duration of sleep are presented in Table 1. Compared to normal sleep duration, those with very short or long duration of sleep were older, less likely to be above primary educated, had longer duration of diabetes, and lower levels of eGFR. In addition, mood-related complaints were higher in those with very short duration and Malays had lower prevalence of normal sleep duration (all *p* < 0.05).

**Table 1:** Demographic and clinical characteristics by sleep duration.

**Characteristics**	**Sleep duration**				
	**Very short, <5 h (*n* = 117, 9.3%)**	**Short, 5 – 6.9 h (*n* = 629, 50.0%)**	**Normal, 7 – 8 h (n = 429, 34.1%)**	**Long, >8 h (*n* = 83, 6.6%)**	***P***
Age, years	66.6 (8.5)	64.2 (9.1)	64.1 (9.1)	67.3 (9.4)	0.001
Gender, Female, %	72 (61.5)	320 (50.9)	202 (47.1)	46 (55.4)	0.04
Ethnicity, Malay, %	40 (34.2)	244 (38.8)	99 (23.1)	20 (24.1)	<0.001
Above primary school education, %	35 (29.9)	251 (39.9)	191 (44.5)	24 (28.9)	0.01
Current smoking, %	13 (11.1)	80 (12.7)	44 (10.3)	10 (12.0)	0.67
Cardiovascular disease, %	24 (20.5)	116 (18.4)	70 (16.3)	20 (24.1)	0.34
Respiratory disorder, %	13 (11.1)	42 (6.7)	22 (5.13)	2 (2.41)	0.048
Mood-related complaints, %	52 (44.4)	247 (39.3)	109 (25.4)	21 (25.3)	<0.001
Duration of diabetes, years	11.4 (9.5)	9.7 (9.2)	9.6 (8.5)	12.7 (13.0)	0.01
Diabetic medication, %	77 (65.8)	416 (66.1)	297 (69.2)	53 (63.9)	0.66
Hypertensive medication use, %	80 (68.4)	385 (61.2)	247 (57.6)	55 (66.3)	0.12
Body mass index, kg/m^2^	27.4 (4.6)	27.4 (5.0)	26.8 (4.5)	27.7 (5.3)	0.19
Obesity, %	79 (67.5)	198 (31.5)	293 (68.3)	64 (77.1)	0.42
Systolic blood pressure, mmHg	140.6 (19.3)	139.9 (18.7)	140.2 (17.9)	143.5 (23.4)	0.43
Hypertension, %	107 (91.5)	525 (83.5)	367 (85.5)	72 (86.7)	0.15
HbA1c, %	7.68 (1.59)	7.58 (1.57)	7.62 (1.49)	7.79 (1.63)	0.66
Total cholesterol, mmol/L	4.89 (1.25)	4.81 (1.23)	4.80 (1.17)	4.89 (1.55)	0.85
eGFR, mL/min/1.73m^2^	70.6 (23.4)	80.0 (22.6)	80.4 (21.1)	70.4 (27.0)	<0.001
UACR^*^, mg/g	141.2 (325.2)	77.5 (247.0)	77.6 (245.3)	85.7 (152.8)	0.27

*eGFR, estimated glomerular filtration rate; HbA1c, hemoglobin A1c; UACR, urinary albumin: creatinine ratio. Data are in mean (SD) or n (%) as appropriate. The Chi-Square Test (for categorical variables) and ANOVA (for continuous variables) were used to compare the demographic and clinical characteristics of participants between the 4 categories of sleep duration*.

* *Indian participants only, where data for UACR was available (n = 781)*.

Associations of sleep duration categories with low eGFR are shown in Table 2. After adjusting for age, gender and ethnicity, as well as for other potential confounders (education, current smoking, cardiovascular disease, respiratory disorders, mood-related complaints, duration of diabetes, diabetic medication, anti-hypertensive medication, obesity, systolic blood pressure, HbA1c and total cholesterol), only long duration of sleep was associated with low eGFR (OR [95% CI] in model 2: 2.31 [1.27–4.19], *P* = 0.01). In Supplementary Table 1, we tested the robustness of this association with sub-analyses stratified by ethnicity. The direction of association between renal insufficiency and long sleep duration was consistent in both Indians and Malays (OR [95% CI] in model 2: 2.07 [0.99–4.30], *p* = 0.05 and 3.81 [1.17–12.37], *p* = 0.03, respectively), although in Indians, the association was only marginally significant.

**Table 2:** Associations of sleep duration with renal insufficiency.

**Sleep duration**	**Renal insufficiency, n (%)**	**Model 1**		**Model 2**	
		**OR (95% CI)**	***P***	**OR (95% CI)**	***P***
Very short, <5 h (*n* = 117)	35 (29.9)	1.39 (0.83–2.32)	0.22	1.22 (0.71–2.09)	0.47
Short, 5–6.9 h (*n* = 629)	118 (18.8)	0.82 (0.58–1.17)	0.27	0.73 (0.51–1.05)	0.09
Normal, 7–8 h (*n* = 429)	81 (18.9)	Reference	–	Reference	–
Long, >8 h (*n* = 83)	34 (41.0)	2.58 (1.48–4.52)	0.001	2.31 (1.27–4.19)	0.01

*Model 1 adjusts for: age, gender, ethnicity. Model 2 adjusts for: Model 1 + education, current smoking, cardiovascular disease, respiratory disorders, mood-related complaints, duration of diabetes, diabetic medication, antihypertensive medication, obesity, systolic blood pressure, HbA1c, total cholesterol*.

Table 3 similarly displays the association of sleep duration categories with albuminuria in Indian participants. In the multivariable model 2, both very short sleep duration (OR [95% CI]: 2.44 [1.36–4.38], P = 0.01) and long sleep duration (OR [95% CI]: 2.37 [1.25–4.50], P = 0.01) were associated with albuminuria. As the presence of low eGFR may influence albuminuria, in a supplementary model (table not shown), we further adjusted for eGFR on top of the co-variates included in model 2—however, results remained similar.

**Table 3:** Associations of sleep duration with albuminuria.

**Sleep duration**	**Albuminuria, n (%)**	**Model 1**		**Model 2**	
		**OR (95% CI)**	***P***	**OR (95% CI)**	***P***
Very short, <5 h (*n* = 66)	32 (48.5)	2.27 (1.31–3.94)	0.01	2.44 (1.36–4.38)	0.01
Short, 5–6.9 h (*n* = 359)	125 (34.8)	1.37 (0.98–1.91)	0.07	1.39 (0.98–1.97)	0.07
Normal, 7–8 h (*n* = 302)	85 (28.1)	Reference	–	Reference	–
Long, >8 h (*n* = 54)	29 (53.7)	2.75 (1.51–4.99)	0.001	2.37 (1.25–4.50)	0.01

*Model 1 adjusts for: age, gender, ethnicity. Model 2 adjusts for: Model 1 + education, current smoking, cardiovascular disease, respiratory disorders, mood-related complaints, duration of diabetes, diabetic medication, antihypertensive medication, obesity, systolic blood pressure, HbA1c, total cholesterol*.

## Discussion

In a population-based sample of Malay and Indian adults with diabetes, long sleep duration (>8 h) was associated with a 2.3 fold higher odds of low eGFR compared to normal sleep duration between 7 and 8 h. Similarly, among Indian participants (where data on UACR was available), both long (>8 h) and very short (<5 h) durations of sleep were associated with 2.4 fold higher odds of albuminuria, respectively. This suggests that abnormally long or short durations of sleep may be associated with DKD.

Literature on the relationship between sleep duration and DKD is scarce (Table 4) (15, 16). In the first of two cross-sectional studies, Ohkuma et al. reported both short and long durations of sleep were associated with higher UACR levels and albuminuria, and long sleep duration was associated with lower eGFR, independent of multiple potential confounders (16). In the second study by Meng et al., where DKD was defined as albuminuria (urinary levels of 24-h microalbumin **>**30 mg/24 h) and/or eGFR (Modification of Diet in Renal Disease equation) <60 mL/ min/1.73 m^2^, short sleep duration was associated with higher multivariable odds of DKD (OR: 1.32, *P* = 0.045); however, the association between long sleep duration and DKD was not significant (OR: 1.83, *P* = 0.10) (15). Our results are therefore in keeping with the notion that abnormally long or short durations of sleep may be associated with greater likelihood of DKD.

**Table 4:** Other studies concerning associations of sleep duration with diabetic or chronic kidney disease.

**1st author, publication year**	**Study design, country**	**Study population size**	**Relevant study outcome(s)**	**Reference sleep duration**	**Results**
**STUDIES OF DIABETIC KIDNEY DISEASE (DKD)**					
Ohkuma et al. (16)	Cross-sectional; Japan	4,870	eGFR [simplified Japanese GFR inference formula equation (28)] Albuminuria and macroalbuminuria, defined as UACR **≥**30 mg/g and **≥**300 mg/g, respectively	6.5 ≤ h <7.5	≥8.5 h was associated with reduced eGFR <4.5 h and ≥7.5 h were associated with albuminuria and higher log-transformed UACR levels <5.5 h and ≥7.5 h were associated with macroalbuminuria
Meng et al. (15)	Cross-sectional; China	1,220	DKD, defined as albuminuria (urinary levels of 24-h microalbumin **>**30 mg/24 h) and/or eGFR [MDRD equation (29)] <60 mL/min/1.73 m^2^	6 ≤ h ≤ 9	<6 h was associated with DKD
**STUDIES OF CHRONIC KIDNEY DISEASE (CKD)**					
Sasaki et al. (30)	Prospective cohort; Japan	3,600	Incident CKD, defined as eGFR [simplified Japanese GFR inference formula equation (28)] <60 mL/min/1.73 m^2^	5 < h < 8	Sleep duration was not associated with risk of CKD. However, 5 ≤ h of sleep was associated with incident CKD in a subgroup of shift workers
McMullan et al. (31)	Prospective cohort; USA	4,238	Incident CKD, defined as eGFR [MDRD equation (29)] <60 mL/min/1.73 m^2^ Rapid decline in eGFR, defined as a decrease in ≥30% eGFR from 1989 to 2000 Albuminuria, defined as UACR ≥30 mg/g	7 ≤ h ≤ 8	5 ≤ h was associated with incident CKD and incident albuminuria 6 ≤ h was associated with rapid decline in eGFR
Yamamoto et al. (32)	Retrospective cohort; Japan	6,834	Incident proteinuria, defined as ≥+1 on the dipstick test	≥7 h	5 ≤ h was associated with incident proteinuria
Salifu et al. (32, 33)	Cross-sectional; USA	128,486	Self-reported CKD	7 h	6 ≤ h and ≥8 h were associated with CKD
Guo et al. (34)	Cross-sectional; China	5,555	Reduced eGFR [CKD-EPI equation (21)], defined as <60 mL/min/1.73 m^2^	7 ≤ h ≤ 8	6 ≤ h was associated with reduced eGFR
Thawornchaisit et al. (35)	Cross-sectional; Thailand	87,143	Self-reported CKD	6 < h <9	6 ≤ h was associated with CKD in men. Sleep duration was not associated with CKD in women
Petrov et al. (36)	Cross-sectional; USA	8,690	UACR, and microalbuminuria defined as UACR ≥30 mg/mmol	7 h	5 ≤ h was associated with microalbuminuria Both shorter and longer durations of sleep were related to increased UACR
Choi et al. (37)	Cross-sectional; South Korea	1,360	Serum creatinine, proteinuria [≥+1 dipstick test), and eGFR (MDRD equation (29)] CKD, defined as either proteinuria or eGFR <60 mL/min/1.73 m^2^	7 ≤ h <8	In women, ≥9 h was associated with high serum creatinine, low eGFR, and CKD in women In men, sleep duration was not associated with serum creatinine, eGFR or CKD
Kim et al. (38)	Cross-sectional; South Korea	241,607	CKD, defined as eGFR [CKD-EPI equation (21)] <60 mL/min/1.73 m^2^ Proteinuria, defined as ≥+1 on the dipstick test	7 h	≥9 h was associated with CKD 5 ≤ h and ≥9 h were associated with proteinuria in female and male subgroups, respectively

*CKD, chronic kidney disease; CKD-EPI, Chronic Kidney Disease Epidemiology Collaboration; DKD, diabetic kidney disease; eGFR, estimated glomerular filtration rate; GFR, glomerular filtration rate; MDRD, Modification of Diet in Renal Disease. In all studies, the exposure variable was self-reported sleep duration*.

Although a few more studies have investigated the relationship between sleep duration and CKD in the general population, results have been inconsistent (Table 4). For instance, in a recent meta-analysis of 6 studies of 252,075 individuals, and 3 studies of 37,197 individuals, to assess the risk of CKD and proteinuria, respectively, in short sleepers, it was found that there was a positive association between short sleep duration and proteinuria, although no significant association was found between short sleep duration and CKD (39). In the meta-analysis, the association between long sleep duration and CKD/proteinuria was not examined for due to limited data (40). However, among the studies that investigated the association of sleep duration with CKD, none of these had had included subgroup analyses by diabetes-status. In our own data, we found that although short and long durations of sleep were associated with higher odds of CKD in the general population, this was largely driven by subjects with diabetes (i.e., there was no association between sleep duration and CKD in SINDI-2 and SIMES-2 participants without diabetes).

The main focus of our study was to investigate if sleep duration was associated with DKD. However, obstructive sleep apnea (OSA) has also been reported as a risk factor for CKD and DKD (40–43). It is hypothesized that the intermittent hypoxia in OSA may lead to increased sympathetic activation, oxidative and nitrosative stress, and impaired microvascular and endothelial regulation in patients with type 2 diabetes, thus resulting in renal dysfunction (44, 45). Additionally, OSA may also affect sleep duration (46). Thus, OSA is a potential confounder as it can influence both sleep duration and DKD. Although the SiMES-2 and SIND-2 epidemiological studies were not designed to formally diagnose OSA [which requires an overnight polysomnogram (46)], the STOP-BANG Questionnaire (47) was included as validated screening tool to detect individuals at high-risk of OSA (defined by a score of ≥4 in this study). As seen in Supplementary Table 2, being at high-risk of OSA was associated with increased odds of renal insufficiency, but not albuminuria. Furthermore, even after adjusting for risk of OSA, the positive associations of long sleep duration with renal insufficiency, and of very short and long sleep durations with albuminuria, remained intact. This suggests that sleep duration is associated higher odds of DKD independent of sleep quality (as may be affected by OSA).

Even though the cross-sectional nature of the current study can neither indicate the direction of causality between short and long durations of sleep with reduced eGFR or albuminuria, nor provide evidence of pathogenic mechanisms that may underlie the reported associations, a few postulations may be discussed. Short and long sleep duration may potentially worsen insulin resistance and glycemic control (48, 49), as well as increase risk of hypertension (50), which are risk factors for DKD itself (10). However, in our study, sleep duration showed no association with HbA1c levels, hypertension, or systolic blood pressure (all *P* > 0.05, table not shown). Furthermore, our results were also adjusted for multiple confounders including glycemic and hypertensive control. Thus, other mechanisms may be contributory. For instance, short and long durations of sleep have also been associated with increased pro-inflammatory markers such as interleukin-6 and C-reactive protein (51, 52), both of which have been implicated in the pathogenesis of DKD (53). However, it is not clear if abnormally short or long sleep durations raise these pro-inflammatory markers, or if these cytokines have sleep-altering qualities themselves (54). Alternatively, a disruption of renal circadian rhythms may potentially lead to DKD. Most renal physiological processes follow a circadian clock, including the regulation of the sodium excretion, the renin-angiotensin system, and blood pressure, which allows the kidney to meet changes in physiological demands throughout a 24-h cycle (55). However, the disruption in sleeping patterns in abnormally short or long sleepers may desynchronise this chronobiological process and predispose toward renal dysfunction. As evidence for this hypothesis, derangement of cyclic behavioral patterns in an animal model resulted in proteinuria, glomerulosclerosis, tubular hyperplasia, and renal fibrosis, consistent with CKD; furthermore, reversal of disease phenotype was seen after controlling for light-dark periodicity to restore circadian rhythms (56).

The results of our study should be considered in light of its limitations. First, habitual sleep duration was self-reported in our study (as opposed to being objectively-measured), which may be subject to misclassification bias (57). Second, we were unable to adjust for other potential confounders such as restless leg syndrome, physical activity levels, and dietary habits, all of which could be linked to both sleep duration and severity of diabetes/DKD (58–60). Third, we only had data on albuminuria in Indian participants, and were thus unable to check the consistency of association between sleep duration and albuminuria in the Malay population. Strengths of our study include a large number of participants with diabetes (*n* = 1,258) from two independent population-based studies with high participation rates (72.1–75.5%), and a standardized assessment of diabetes, eGFR, albuminuria, and other associated systemic risk factors.

In summary, we found that the overall odds of low eGFR was more than double in those with long sleep duration compared to participants with a normal sleep duration of 7–8 h. Similarly, in Indians (with data on albuminuria), the odds of albuminuria associated with either short and long sleep duration was more than 2 fold compared to normal. Alongside other reports associating abnormal durations of sleep with a multitude of adverse health outcomes [e.g., cardiovascular disease (61), diabetes (13), and all-cause mortality (62)], our study suggests that habitual sleep duration may be a modifiable risk factor for CKD in patients with diabetes. However, longitudinal studies on sleep duration and DKD are lacking, and would be required for confirmation and translating these findings into prevention and care.

## Data Availability

As the study involves human participants, the data cannot be made freely available in the manuscript, the supplemental files, or a public repository due to ethical restrictions. Nevertheless, the data are available from the Singapore Eye Research Institutional Ethics Committee for researchers who meet the criteria for access to confidential data.

## Author Contributions

All authors contributed to the intellectual development of this paper. CS and NT designed the study. NT performed the statistical analyses, wrote and revised the initial draft. JC assisted with the initial draft. CS supervised data analysis. TW obtained funding. JC, C-YC, TW, and CS provided critical corrections to the manuscript. Final version of the paper was read and approved by all the authors.

### Conflict of Interest Statement

The authors declare that the research was conducted in the absence of any commercial or financial relationships that could be construed as a potential conflict of interest.
