# Supplementary material for: Sleep Duration and Diabetic Kidney Disease
Source: Front Endocrinol (Lausanne). 2019 Jan 14;9:808. doi: 10.3389/fendo.2018.00808 (PMC6340267; doi:10.3389/fendo.2018.00808)
Supplement: Supplementary file 1 [file Table_1.DOCX]

| Supplementary Table 1. Associations of Sleep Duration with Renal Insufficiency, Stratified by Ethnicity. | | | | | |
| --- | --- | --- | --- | --- | --- |
|  | | Model 1 | | Model 2 | |
| Sleep duration | **Renal insufficiency, n (%)** | **OR (95% CI)** | **P** | **OR (95% CI)** | **P** |
| Indian (*n* = 855) | | | | | |
| Very short, <5 h (*n* = 77) | 16 (20.8) | 1.28 (0.64 – 2.54) | 0.49 | 1.25 (0.62 – 2.54) | 0.54 |
| Short, 5 - 6.9 h (*n* = 385) | 49 (12.7) | 0.86 (0.54 – 1.36) | 0.51 | 0.80 (0.49 – 1.29) | 0.36 |
| Normal, 7 - 8 h (*n* = 330) | 49 (14.8) | Reference | - | Reference | - |
| Long, >8 h (*n* = 63) | 21 (33.3) | 2.15 (1.09 – 4.24) | 0.03 | 2.07 (0.99 – 4.30) | 0.05 |
| Malay (*n* = 403) | | | | | |
| Very short, <5 h (*n* = 40) | 19 (47.5) | 1.57 (0.71 – 3.50) | 0.27 | 1.10 (0.45 – 2.66) | 0.84 |
| Short, 5 - 6.9 h (*n* = 244) | 69 (28.3) | 0.81 (0.47 – 1.40) | 0.45 | 0.66 (0.37 – 1.19) | 0.17 |
| Normal, 7 - 8 h (*n* = 99) | 32 (32.3) | Reference | - | Reference | - |
| Long, >8 h (*n* = 20) | 13 (65.0) | 4.01 (1.36 – 11.87) | 0.01 | 3.81 (1.17 – 12.37) | 0.03 |
| Model 1 adjusts for: age, gender, ethnicity.  Model 2 adjusts for: Model 1 + education, current smoking, cardiovascular disease, respiratory disorders, mood-related complaints, duration of diabetes, diabetic medication, antihypertensive medication, obesity, systolic blood pressure, HbA1c, total cholesterol. | | | | | |

| Supplementary Table 2. Associations of Obstructive Sleep Apnea with Renal Insufficiency and Albuminuria. | | | | | | |
| --- | --- | --- | --- | --- | --- | --- |
|  | Renal insufficiency | | | Albuminuria* | | |
| Sleep parameter | ***n* (%)** | **OR (95% CI)** | **P** | ***n* (%)** | **OR (95% CI)** | **P** |
| Risk of OSA† |  |  |  |  |  |  |
| Yes (*n* = 62 Malay, 183 Indian) | 64 (26.1) | 1.58 (1.03 – 2.40) | 0.001 | 59 (34.3) | 0.84 (0.55 – 1.28) | 0.41 |
| No (*n* = 341 Malay, 672 Indian) | 204 (20.1) | Reference | - | 212 (34.8) | Reference | - |
| Sleep duration |  |  |  |  |  |  |
| Very short, <5 h (*n* = 40 Malay, 77 Indian) | 35 (29.9) | 1.24 (0.72 – 2.12) | 0.44 | 32 (48.5) | 2.45 (1.37 – 4.40) | 0.01 |
| Short, 5 - 6.9 h (*n* = 244 Malay, 385 Indian) | 118 (18.8) | 0.70 (0.49 – 1.02) | 0.06 | 125 (34.8) | 1.40 (0.98 – 1.98) | 0.06 |
| Normal, 7 - 8 h (*n* = 99 Malay, 330 Indian) | 81 (18.9) | Reference | - | 85 (28.1) | Reference | - |
| Long, >8 h (*n* = 20 Malay, 63 Indian) | 34 (41.0) | 2.24 (1.23 – 4.08) | 0.01 | 29 (53.7) | 2.41 (1.27 – 4.57) | 0.01 |
| OSA = Obstructive Sleep Apnea.  The models adjust for the co-variables age, gender, ethnicity, education, current smoking, cardiovascular disease, respiratory disorders, mood-related complaints, duration of diabetes, diabetic medication, antihypertensive medication, obesity, systolic blood pressure, HbA1c, total cholesterol; in addition, risk of OSA and sleep duration are adjusted for each other.  *Results for the outcome of albuminuria are presented for Indian participants with data on urinary creatinine and albumin (*n* = 781) only.  †Risk for OSA was defined as a STOP-Bang score of ≥ 4. | | | | | | |
